# Polyglutamine Toxicity Is Controlled by Prion Composition and Gene Dosage in Yeast

**DOI:** 10.1371/journal.pgen.1002634

**Published:** 2012-04-19

**Authors:** He Gong, Nina V. Romanova, Kim D. Allen, Pavithra Chandramowlishwaran, Kavita Gokhale, Gary P. Newnam, Piotr Mieczkowski, Michael Y. Sherman, Yury O. Chernoff

**Affiliations:** 1 School of Biology, Georgia Institute of Technology, Atlanta, Georgia, United States of America; 2 School of Medicine, The University of North Carolina at Chapel Hill, Chapel Hill, North Carolina, United States of America; 3 Department of Biochemistry, Boston University School of Medicine, Boston, Massachusetts, United States of America; Brown University, United States of America

## Abstract

Polyglutamine expansion causes diseases in humans and other mammals. One example is Huntington's disease. Fragments of human huntingtin protein having an expanded polyglutamine stretch form aggregates and cause cytotoxicity in yeast cells bearing endogenous QN-rich proteins in the aggregated (prion) form. Attachment of the proline(P)-rich region targets polyglutamines to the large perinuclear deposit (aggresome). Aggresome formation ameliorates polyglutamine cytotoxicity in cells containing only the prion form of Rnq1 protein. Here we show that expanded polyglutamines both with (poly-QP) or without (poly-Q) a P-rich stretch remain toxic in the presence of the prion form of translation termination (release) factor Sup35 (eRF3). A Sup35 derivative that lacks the QN-rich domain and is unable to be incorporated into aggregates counteracts cytotoxicity, suggesting that toxicity is due to Sup35 sequestration. Increase in the levels of another release factor, Sup45 (eRF1), due to either disomy by chromosome II containing the *SUP45* gene or to introduction of the *SUP45*-bearing plasmid counteracts poly-Q or poly-QP toxicity in the presence of the Sup35 prion. Protein analysis confirms that polyglutamines alter aggregation patterns of Sup35 and promote aggregation of Sup45, while excess Sup45 counteracts these effects. Our data show that one and the same mode of polyglutamine aggregation could be cytoprotective or cytotoxic, depending on the composition of other aggregates in a eukaryotic cell, and demonstrate that other aggregates expand the range of proteins that are susceptible to sequestration by polyglutamines.

## Author Summary

Polyglutamine diseases, including Huntington disease, are associated with expansions of polyglutamine tracts, resulting in aggregation of respective proteins. The severity of Huntington disease is controlled by both DNA and non–DNA factors. Mechanisms of such a control are poorly understood. Polyglutamine may sequester other cellular proteins; however, different experimental models have pointed to different sequestered proteins. By using a yeast model, we demonstrate that the mechanism of polyglutamine toxicity is driven by the composition of other (endogenous) aggregates (for example, yeast prions) present in a eukaryotic cell. Although these aggregates do not necessarily cause significant toxicity on their own, they serve as mediators in protein sequestration and therefore determine which specific proteins are to be sequestered by polyglutamines. We also show that polyglutamine deposition into an aggresome, a perinuclear compartment thought to be cytoprotective, fails to ameliorate cytotoxicity in cells with certain compositions of pre-existing aggregates. Finally, we demonstrate that an increase in the dosage of a sequestered protein due to aneuploidy by a chromosome carrying a respective gene may rescue cytotoxicity. Our data shed light on genetic and epigenetic mechanisms modulating polyglutamine cytotoxicity and establish a new approach for identifying potential therapeutic targets through characterization of the endogenous aggregated proteins.

## Introduction

A variety of human neurodegenerative disorders are associated with expansions of polyglutamine (poly-Q) repeats in certain proteins [1], [2]. One well known example is Huntington's disease (HD), which is caused by an expansion of the poly-Q stretch, located within the N-terminal stretch of the essential protein called huntingtin (Htt) [3]. Poly-Q expansion promotes formation of aggregates by the proteolytic Htt fragments containing an expanded poly-Q stretch [4], [5]. As the poly-Q-expanded N-terminal region of Htt is shown to aggregate and produce HD-like neurodegeneration in the mouse model, it is clear that this region is sufficient for reproducing the characteristic features of poly-Q aggregation and toxicity [6], [7]. Poly-Q associated pathologies can not be explained solely by the loss of the cellular function of a respective protein, *e. g.* Htt (for review, see [1]). Sequestration of other essential proteins by poly-Q aggregates was proposed to be a possible mechanism for toxicity [1], [8]. However, different experimental models suggested different candidates for sequestration [9]–[12], which decreased enthusiasm for the sequestration model.

To complicate matters further, expanded poly-Q proteins form various types of aggregates in mammalian cells [13], [14]. In the case of Htt, both nuclear and cytoplasmic aggregates were found [4], [15], [16]. Their contributions to poly-Q pathogenicity remain a topic of intense discussion [17], [18]. At least, most researchers agree that one type of cytoplasmic aggregated structure, so-called “aggresome”, plays a cytoprotective role via assembling poly-Q expanded Htt at one site and possibly promoting its autophagy-dependent clearance [19]–[22]. The aggresome is located perinuclearly, associated with the centrosome, and assembled with participation of the microtubular cytoskeleton. Other misfolded proteins can also be sequestered into an aggresome, indicating that this structure serves as a universal quality control depot for aggregating proteins [19], [23]–[27].

Experimental assays for studying the molecular mechanism of poly-Q aggregation and toxicity have been developed in the yeast *Saccharomyces cerevisiae* [28]–[34]. It has been shown that cytoplasmic aggregation and toxicity of the chimeric protein, generated by a fusion of the expanded poly-Q stretch of Htt to the green fluorescent protein (GFP), is facilitated by the presence of the endogenous yeast prions, [*PIN^+^*] and/or [*PSI^+^*] [30], [35]. In the absence of a prion, aggregates of this construct were rare, and no significant cytotoxicity was detected. However, in the presence of a prion, multiple peripherally located aggregates were formed, and cytotoxicity was observed [30]. The prions [*PIN^+^*] and [*PSI^+^*] are self-perpetuating aggregates of the endogenous yeast proteins Rnq1 (unknown function) and Sup35 (translation termination, or release factor, also called eRF1), respectively (for review, see [36]). Both of these proteins contain QN-rich prion domains (PrDs) that are responsible for aggregation properties (for review, see [37], [38]). It is likely that pre-existing prion aggregates nucleate aggregation of poly-Q expanded huntingtin. In the case of the Rnq1 prion, it was shown that poly-Q aggregates sequester some cytoskeletal components and inhibit endocytosis, which apparently contributes to cytotoxicity [39]. Inhibition of endocytosis was also detected in mammalian cells expressing poly-Q [40]. As mammalian Htt has been proposed to play a role in vesicle trafficking [41], these results are likely relevant to human HD.

Flanking sequences modulate poly-Q toxicity [32], [42]. In yeast strains containing the Rnq1 prion, cytotoxicity was eliminated by using a longer Htt fragment, which includes a proline (P)-rich stretch in addition to poly-Q. This P-rich stretch was shown to target aggregated poly-Q protein into a single perinuclear microtubule-dependent deposit, co-localized with the spindle body (yeast counterpart of a centrosome) and therefore resembling a mammalian aggresome [42]. The cytoprotective role of the aggresome, as opposed to cytotoxicity of some other types of aggregates, recapitulates the situation previously observed in mammalian cells [20], [21], [23].

While the prion form of Sup35 protein ([*PSI^+^*]) also promotes poly-Q toxicity in the yeast assay [35], the mechanism for this toxicity has not been studied in detail previously. In our current work, we demonstrate that [*PSI^+^*]-dependent poly-Q toxicity is not counteracted by aggresome formation, but is ameliorated by an increased dosage of some components of the translational termination machinery. These data show that targets of poly-Q toxicity and the cytoprotective potential of the aggresome depend on the composition of endogenous aggregated proteins in a eukaryotic cell.

## Results

### Aggresome formation does not ameliorate polyglutamine toxicity in the presence of Sup35 prion

**Figure 1:**
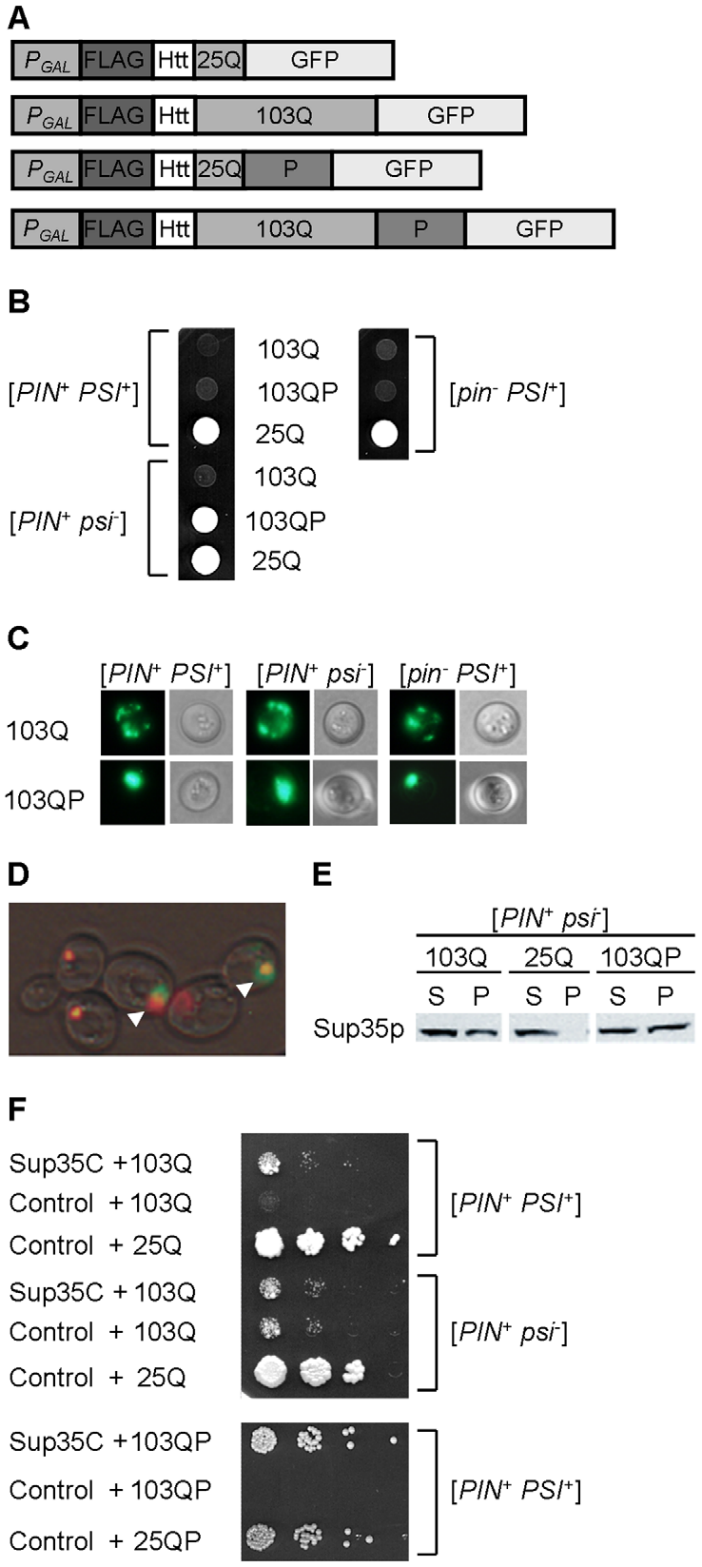
Polyglutamine toxicity and aggregation in the yeast strains with various prion compositions. A – Polyglutamine constructs used in this work. All constructs were under the control of the galactose-inducible promoter (*P_GAL_*), and contained the FLAG epitope, N-terminal 17 amino acid residues and poly-Q stretch of human Htt, and were fused to the gene coding for green fluorescent protein (GFP) at C-terminus. Numbers indicate length of poly-Q stretch. Poly-QP constructs also contained the proline-rich region of Htt (designated as P), immediately following the poly-Q stretch. B – Expanded poly-Q without a P-rich region (103Q), expressed under the *P_GAL_* promoter on -Ura/Gal medium, is toxic in the presence of either [*PIN* ^+^] or [*PSI* ^+^] (or both), with two prions showing an additive effect. In contrast, expanded poly-Q with a P-rich region (103QP) is toxic only in the presence of [*PSI* ^+^]. The 25Q construct, not exhibiting toxicity under these conditions, is shown as a control. The 25QP construct (not shown) behaved in the same way as 25Q. C – 103Q and 103QP form multiple peripheral aggregates and single aggregate (aggresome), respectively, in cells containing either or both prions ([*PIN^+^*] and/or [*PSI^+^*]), as visualized by fluorescence microscopy. Perinuclear location of aggresome (not shown) was confirmed by DAPI staining as described previously [42]. D – Overexpressed Sup35NM-DsRed (red) forms large clumps in the [*PSI^+^*] cells, that overlap with the 103QP-GFP aggresome (green), as pointed by arrows. E - Expression of 103Q or 103QP promotes aggregation of Sup35 in the [*psi^−^*] strain as seen by an increase of pellet (P) versus supernatant (S) fraction, in comparison to the respective strain expressing 25Q. Centrifugation analysis was followed by Western blotting and immunostaining with the Sup35 antibody. F - Expression of the Sup35 derivative, lacking the prion and middle domains (Sup35C), decreases 103Q and 103QP toxicity in the [*PIN^+^ PSI^+^*] strain but does not influence 103Q toxicity in the [*PIN^+^ psi^−^*] strain. *SUP35C* gene was under control of the endogenous *SUP35* promoter. Serial decimal dilutions were spotted onto -Ura/Gal medium.

To distinguish between the different patterns of poly-Q aggregation in yeast, we have employed the previously described constructs (Figure 1A) that produce the N-proximal region of Htt, fused to the FLAG epitope at the N-terminus and the green fluorescent protein (GFP) at the C-terminus. The N-terminal Htt region included the poly-Q stretch, which is either followed (poly-QP) or not followed (poly-Q) by the P-rich region. The poly-Q expanded versions (103Q and 103QP) contained a stretch of 103 glutamine residues, which corresponds to a severe form of Huntington's disease, while control non-aggregating versions (25Q and 25QP) contained 25 glutamine residues. As there was no difference in the effects of 25Q and 25QP, some of the figures show only the 25Q control. As described previously [30], the 103Q construct was toxic to yeast strains containing either [*PIN^+^*] (Rnq1 protein in a prion form) or [*PSI^+^*] (Sup35 protein in a prion form), with a combination of both prions showing an additive effect (Figure 1B). Also in agreement with previous observations [42], the 103QP construct was not toxic to the strains containing only Rnq1 prion. Surprisingly, the 103QP construct was toxic to the strains containing the Sup35 prion, independently in the presence or absence of Rnq1 prion (Figure 1B). Fluorescence microscopy confirmed that 103QP preferentially formed a single perinuclear aggregate deposit (aggresome) in the cells containing Rnq1 and/or Sup35 prions, while 103Q produced multiple peripheral aggregates (Figure 1C). Therefore, the ability of poly-QP to form an aggresome was not affected by the Sup35 prion, however, amelioration of toxicity by the aggresome was impaired. These data show that the mechanism of polyglutamine toxicity, promoted by the Sup35 prion, is different from the mechanism of polyglutamine toxicity promoted by the Rnq1 prion. Unless stated otherwise, all further experiments were performed in the strains containing the [*PIN* ^+^] prion, thereby comparing the [*PSI* ^+^] and [*psi* ^−^] derivatives so that we could distinguish the effects attributed specifically to the [*PSI* ^+^] prion.

### Polyglutamine accumulation leads to Sup35 sequestration

Notably, when Sup35NM, tagged with DsRed, and 103QP-GFP are co-overproduced in the [*PSI^+^*] strain, most of the Sup35NM-DsRed is eventually assembled into one large deposit, that is either partially or completely overlapping with the 103QP aggresome (Figure 1D). This indicates a possibility of sequestration of Sup35 by the aggresome, and agrees with the previous observation [43], confirmed by us (Figure 1E) that polyglutamines promote aggregation of a fraction of Sup35, even in a [*psi^−^*] strain. Notably, 103QP toxicity in the [*PSI^+^*] cells was ameliorated by introducing the Sup35 derivative (designated Sup35C) that lacks the N-terminal (prion) and middle domains and therefore, is functional but unable to be incorporated into the prion aggregates (Figure 1F). Expression of Sup35C also decreased toxicity of 103Q in the [*PIN^+^ PSI^+^*] strain down to the levels observed in the [*PIN^+^ psi^−^*] strain. However, Sup35C did not affect toxicity of 103Q in [*PIN^+^ psi* ^−^]. These data confirm that in the [*PSI^+^*] strain (but not in the [*PIN^+^ psi^−^*] strain), sequestration of Sup35 contributes to polyglutamine toxicity.

### Isolation of the anti-polyQ-toxic (*AQT*) mutants

**Figure 2:**
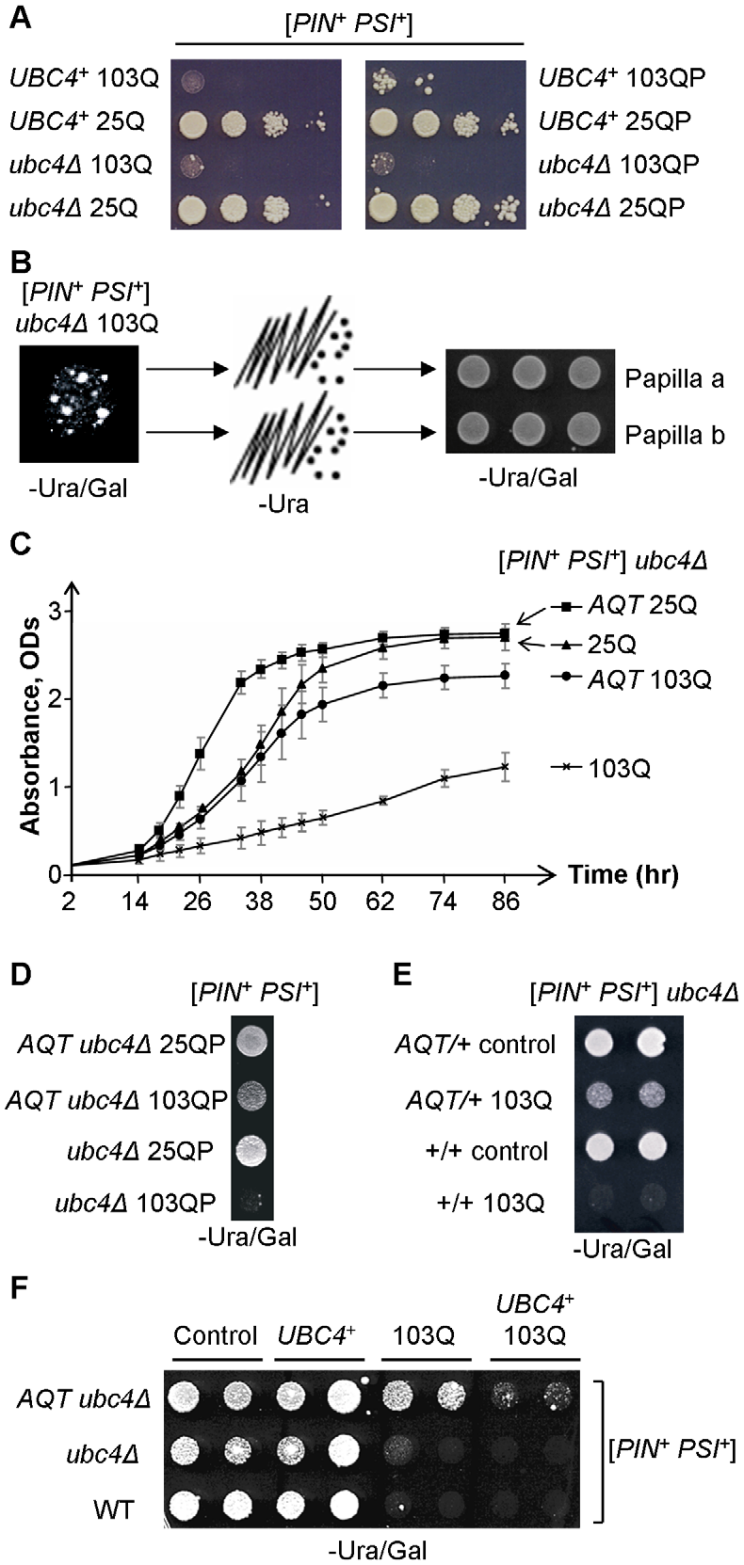
Isolation and characterization of anti-polyQ toxicity (*AQT*) derivatives. A – *Ubc4*Δ has no significant effect on toxicity of 103Q or 103QP in the [*PIN* ^+^ *PSI* ^+^] background. Serial decimal dilutions were spotted onto -Ura/Gal medium. B – Papillae arise spontaneously in the *ubc4*Δ [*PIN* ^+^ *PSI* ^+^] strain expressing 103Q, and are able to stably maintain the anti-polyQ-toxic phenotype after colony purification. These papillae were designated as *AQT*. C – Comparison of the growth curves of [*PIN^+^ PSI^+^*] *ubc4*Δ strains that differ by polyglutamine constructs and by the presence or absence of *AQT*. Growth was measured by optical density at 600 nm in the liquid –Ura medium with galactose and raffinose instead of glucose. At least 3 independent cultures were characterized per each combination. Error bars represent standard deviations. D – *AQT* ameliorates 103QP toxicity. E – *AQT* is dominant (all strains are [*PIN^+^ PSI^+^*] and *ubc4*Δ homozygotes). F – Reintroduction of the *UBC4* gene under galactose-inducible promoter on a multicopy plasmid partly suppresses but does not completely eliminate anti-toxic effect of *AQT*. -Ura/Gal plates are scored on panels D, E and F.

Next, we looked for other genetic factors influencing the [*PSI* ^+^]-dependent polyglutamine toxicity. As the ubiquitin-proteasome system (UPS) is known to influence poly-Q effects in mammalian cells, we have studied poly-Q toxicity in the yeast strain with a deletion of the *UBC4* gene, coding for one of the major yeast ubiquitin-conjugating enzymes [44]. *Ubc4*Δ did not improve growth in the presence of polyglutamines (Figure 2A) however the *ubc4Δ 103Q* strain produced spontaneously arising fast-growing papillae (Figure 2B). Three independent papillae were analyzed further, and each was confirmed to stably reproduce the anti-toxic phenotype (Figure 2B and 2C), and also to ameliorate toxicity of 103QP (Figure 2D). These derivatives were named *AQT* for *A*nti-poly*Q T*oxicity, with respective phenotype designated as Aqt^+^. Specific effect of *AQT* on toxicity caused by expanded polyglutamines was especially pronounced after longer periods of incubation (Figure 2C). All *AQT* derivatives retained the [*PIN^+^*] and [*PSI^+^*] prions (data not shown). In each derivative, the Aqt^+^ phenotype was dominant (see Figure 2E as an example) and segregated in a Mendelian fashion in meiosis (Table S1). All pairwise genetic crosses between three independent *AQT* derivatives produced 4 *AQT*: 0 wild-type pattern of segregation in the vast majority of tetrads (Table S2), indicating that all *AQT* derivatives are formally confined to a single genetic locus. Reintroduction of the wild-type *UBC4* gene into the *AQT* strain decreased but did not completely eliminate amelioration of toxicity, indicating that *ubc4Δ* strengthens the Aqt^+^ phenotype but is not required for its manifestation (Figure 2F).

**Figure 3:**
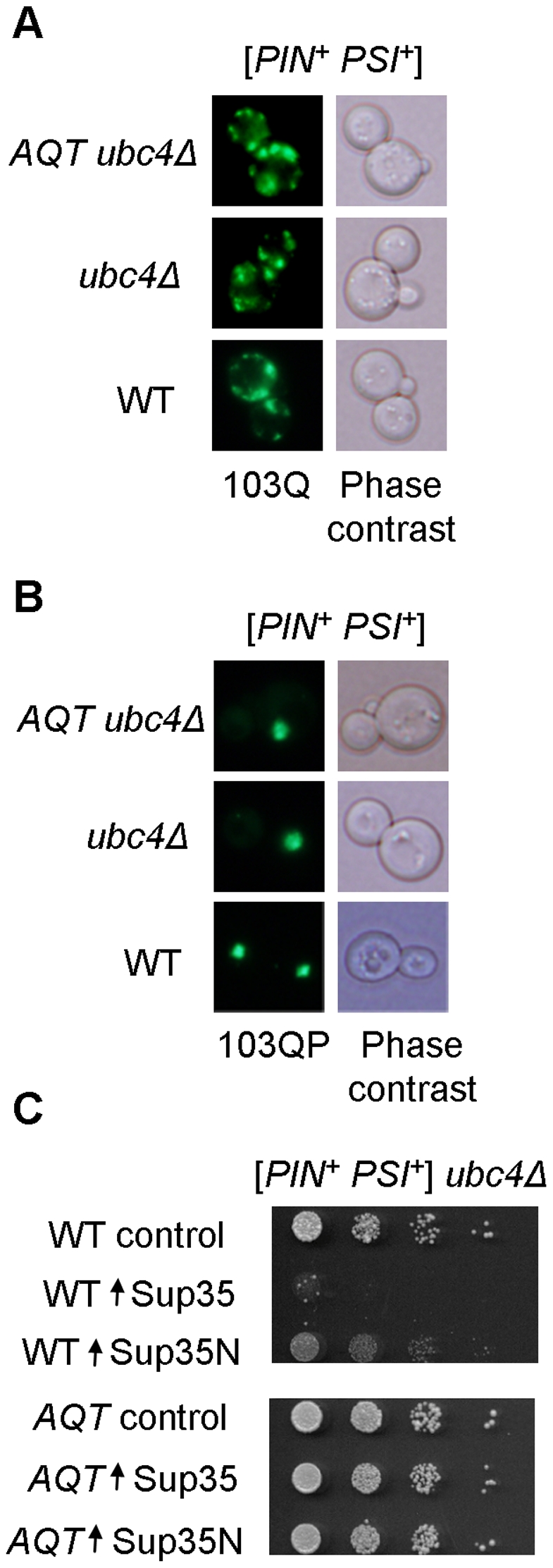
Effects of *AQT* on polyglutamine aggregation and Sup35 toxicity. A and B – Typical aggregation patterns of 103Q (multiple dots, A) and 103QP (single clump, B) are not affected by *AQT*, as confirmed by fluorescence microscopy. C – *AQT* mutant ameliorates toxicity of excess Sup35 or Sup35N in the [*PSI* ^+^] strain. Sup35 and Sup35N proteins were expressed from centromeric plasmid under control of the galactose-inducible promoter. Cells were grown on the -Ura/glucose medium selective for the plasmid for 1 day. Serial decimal dilutions were plated onto -Ura/Gal medium.

Despite their anti-toxic effect, *AQT* derivatives retained the typical mode of cytologically detectable aggregation for both 103Q (multiple peripheral aggregates, Figure 3A) and 103QP (single perinuclear aggregate, Figure 3B), indicating that amelioration of toxicity is not due to a lack of aggregation. Overproduction of Sup35 protein or its prion domain, Sup35N, is known to inhibit growth of the [*PSI^+^*] strains [37], [38]. This effect was ameliorated in *AQT* derivatives (Figure 3C). The *AQT* strains also exhibited additional phenotypes that were not directly related to amelioration of toxicity, such as compensation of the *ubc4Δ*-mediated temperature sensitivity and loss of the invasive growth capability (Figure S1). *AQT* also slightly increased the growth of the [*PIN^+^ psi^−^*] *ubc4Δ* strain expressing 103Q, especially after shorter incubation periods (Figure S1D), possibly due to increased robustness of the *AQT ubc4Δ* strain in the stressful conditions.

### *AQT* derivatives are disomics of chromosome II

**Figure 4:**
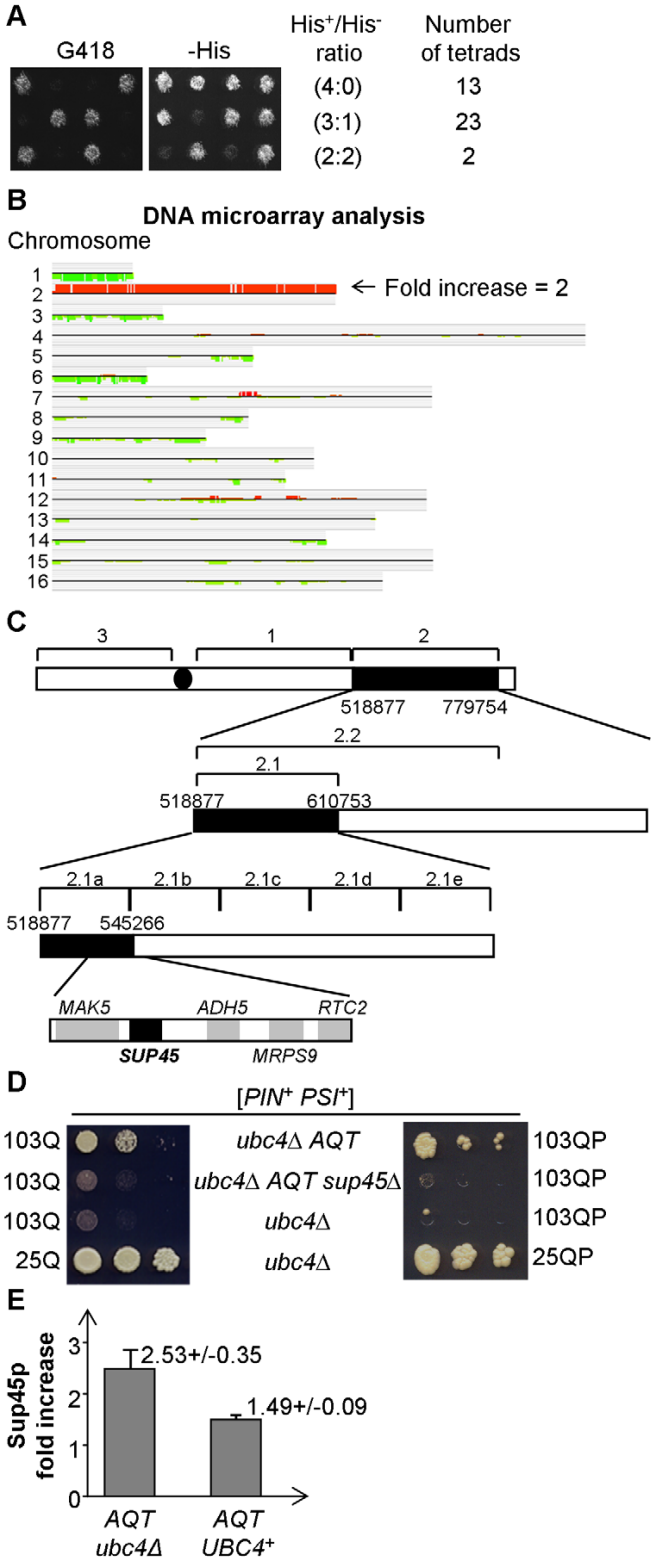
*AQT* derivatives are disomic for chromosome II, and extra-copy of *SUP45* is responsible for antitoxicity. A – Tetrad analysis of a diploid obtained from mating of the *AQT* strain bearing the *ubc4*Δ*::HIS3* transplacement, to the strain bearing the *ubc4*Δ*::KanMX* transplacement, demonstrates presence of at least 2 copies of the *HIS3* gene versus one copy of the *KanMX* gene. This can be concluded from the fact that majority of tetrads produce more than 2 His^+^ spores, in contrast to the typically 2∶2 segregation by G418 resistance caused by KanMX. All *AQT* spores in this cross were His^+^ (not shown). B – Hybridization of total DNA to a complete DNA microarray of the *S. cerevisiae* genome confirms that all the coding material of chromosome II is duplicated in the *AQT* strain. Comparison is performed according to CLAC (CLuster Along Chromosome) consensus plot. For procedure, see Text S1. C – Sequential deletion mapping of the chromosome II extra copy in the *AQT* strain. The *AQT#7* derivative (see Figure S2B) was used in these experiments. Each numbered region corresponds to a respective deletion. Deletions eliminating the antitoxicity phenotype in the [*PSI^+^*] background are shown as boxes filled in black. All deletions were verified by PCR. Five ORFs located within region 2.1a were each deleted individually; among those deletions, only deletion of *SUP45* eliminated *AQT* as shown on panels B and C. D – Elimination of the antitoxic effect on 103Q and 103QP by the *sup45* deletion in *AQT* strain. Serial decimal dilutions were spotted onto -Ura/Gal medium. E – Sup45 protein levels are elevated in the *AQT* strain, more profoundly in *ubc4*Δ background than in the presence of wild type *UBC4* gene (*UBC4^+^*). Sup45p level is shown relative to the isogenic monosomic (non-*AQT*) control in each case. Ade2 protein was used as the loading control. At least 3 measurements with independent cultures were performed in each case. Error bars correspond to standard deviations. In each case the difference in Sup45 levels between the *AQT* and non-*AQT* strain is statistically significant as confidence limits do not overlap, and differences between the *UBC4^+^* and *ubc4Δ* strains are statistically significant according to *t*-criterium (*P_Ho_*<0.01).

Genetic crosses aimed at characterizing the inheritance of *AQT* revealed that *AQT* is centromere-linked (Figure S2A). Moreover, when the original *AQT* derivative, obtained in the strain bearing the *ubc4Δ::HIS3* disruption was mated to the wild-type strain bearing the *ubc4Δ::KanMX* disruption (causing resistance to the antibiotic G418), both *AQT* and *KanMX* markers segregated 2∶2 in meiosis as expected, while majority of tetrads did show 3∶1 or 4∶0 segregation for the His^+^ phenotype, indicative of the presence of two copies of the *HIS3* allele in the cross (Figure 4A). Notably, all *AQT* spores (76 total) obtained from tetrads with a 2∶2 ratio for *KanMX* were His^+^. The simplest scenario compatible with these ratios is that the *AQT* derivative is a disomic of chromosome II, where the *ubc4Δ:: HIS3* allele is located. As chromosome segregation is controlled by the centromere, this would also explain the centromere linkage of *AQT*. Indeed, fractionation of the yeast chromosomes via CHEF (Contour-clamped Homogeneous Electric Field) gel electrophoresis confirmed that each of the three independent *AQT* derivatives contains an additional copy of the chromosome II band (Figure S2B). Extra-copy of chromosome II also co-segregated with *AQT* in tetrad analysis (data not shown). Microarray-based analysis of genomic DNA of the three original *AQT* derivatives and two *AQT* meiotic segregants confirmed that each of these strains contains an extra copy of every piece of the coding material in chromosome II (Figure 4B). Overall, our data demonstrate that *AQT* is associated with an extra copy of chromosome II.

### Extra copy of the *SUP45* gene is responsible for the amelioration of [*PSI^+^*]-dependent polyglutamine toxicity in the *AQT* derivatives

Sequential deletion analysis of the extra copy of chromosome II in the *AQT* strain confined the gene responsible for amelioration of toxicity to the region of the right arm, located between positions 528161 and 537490 (Figure 4C). This region contains 5 ORFs, including the essential gene *SUP45*, that codes for a translation termination factor Sup45, or eRF1, working together with Sup35 (eRF3) [45]. We have disrupted the copy of the *SUP45* gene, located on the duplicated chromosome II in the *AQT* strain, and have shown that this disruption eliminates the anti-toxicity effect on both 103Q and 103QP (Figure 4D). Notably, other phenotypes, associated with chromosome II disomy but not related to amelioration of polyglutamine toxicity in the [*PSI* ^+^] strain, including slightly increased growth of the [*PIN^+^ psi^−^*] strain in the presence of 103Q, were not affected by *sup45Δ* (Figure S1B, S1C and S1E).

Western blot analysis confirmed that the *AQT* derivative contains more Sup45 protein, compared to the isogenic wild type strain (Figure 4E). This increase was more profound in the *ubc4Δ* than in the *UBC4* ^+^ background. This explains why the *AQT* effect was better seen in *ubc4Δ*. Thus, an increase in Sup45 levels due to the presence of an extra-copy of the *SUP45* gene is responsible for the anti-toxic (Aqt^+^) phenotype in the [*PSI^+^*] background.

### Plasmid-mediated overproduction of a release factor also ameliorates polyglutamine toxicity

**Figure 5:**
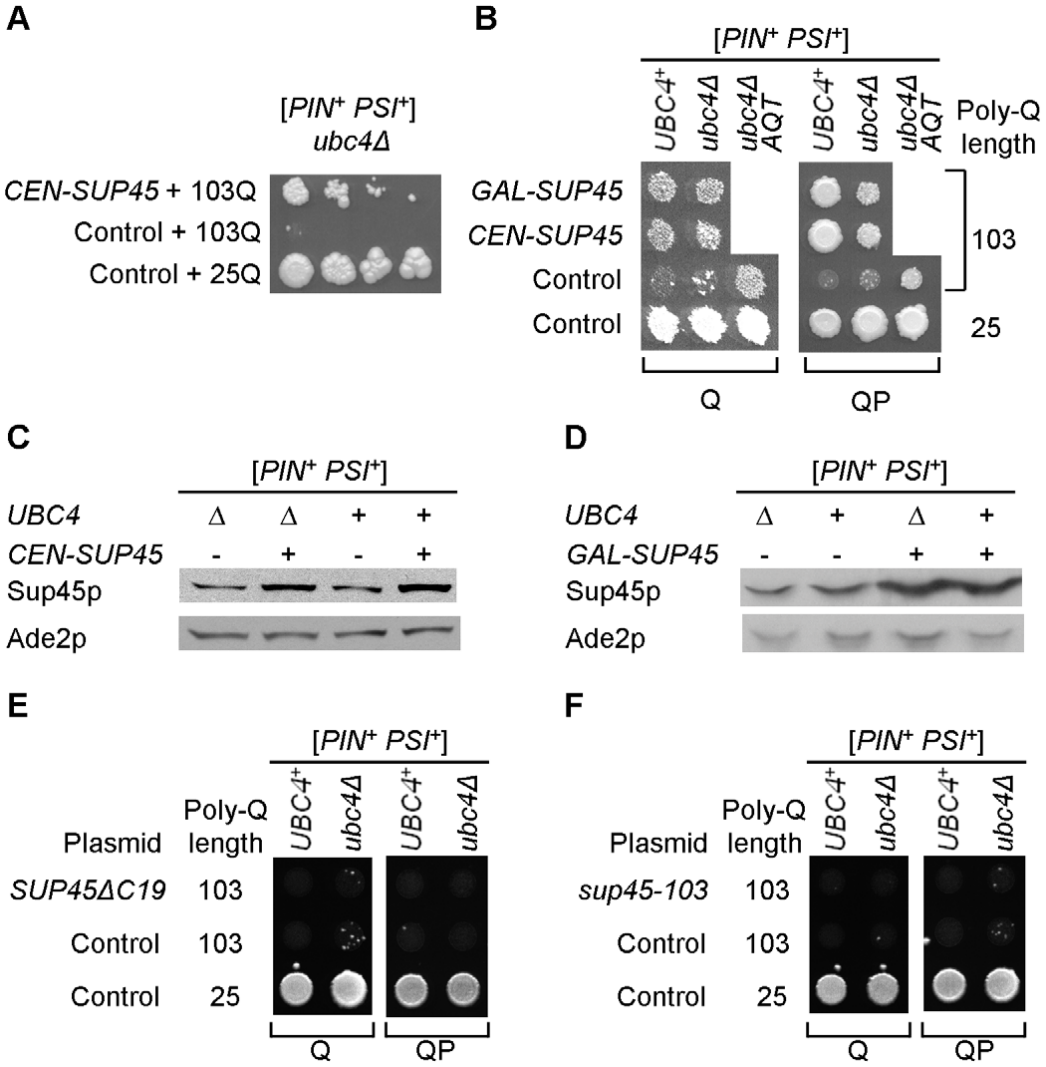
Modulation of polyglutamine toxicity by the plasmid-borne release factor genes. A - An extra copy of *SUP45* gene, located on the centromeric plasmid under endogenous promoter, ameliorates toxicity of 103Q in the *ubc4*Δ [*PSI^+^*] strain, as seen from serial decimal dilutions plated onto the galactose medium selective for both poly-Q and *SUP45* (or control) plasmids. B – Amelioration of [*PSI^+^*]-dependent polyglutamine toxicity by a plasmid-borne extra copy of *SUP45* gene is detected for both endogenous (*CEN-SUP45*) galactose-inducible (*GAL-SUP45*) promoters, for both 103Q and 103QP constructs, and in both *ubc4*Δ and *UBC4^+^* strains. Antitoxic effect of the plasmid-borne *SUP45* gene in the *ubc4Δ* strain is comparable to antitoxic effect of *AQT*. Toxicity was scored on the galactose medium selective for both poly-Q and *SUP45* (or control) plasmids. C and D – Centromeric plasmids with *SUP45* gene under endogenous (C) or galactose-inducible *P_GAL_* (D) promoters increase levels of Sup45 protein (Sup45p) both [*UBC4^+^*] and *ubc4*Δ strains. Cultures were grown in liquid -Ura -Leu glucose (C) or -Ura -Leu galactose/raffinose (D) medium. Ade2 (Ade2p) protein is shown as a loading control. E and F – Plasmids, expressing the *SUP45* alleles with either C-terminal deletion, *SUP45*Δ*C19* (that abolishes Sup45 function and interaction with Sup35) (E) or missense mutation *sup45-103*, T62C (that impairs Sup45 function) (F) from the endogenous *SUP45* promoter, do not ameliorate 103Q and 103QP toxicity, as scored on the galactose medium selective for both plasmids.

Next, we checked if an increase in the Sup45 levels, produced by means other than duplication of chromosome II, would also ameliorate the [*PSI^+^*]-dependent polyglutamine toxicity. Indeed, introduction of the centromeric plasmid bearing the *SUP45* gene under its own (Figure 5A) or galactose-inducible (Figure 5B) promoter (in the latter case, under inducing conditions) ameliorated toxicity of both 103Q and 103QP. Anti-toxic effect of plasmid-borne *SUP45* was clearly detected in both *ubc4*Δ and *UBC4^+^* backgrounds, indicating that it is less sensitive to the presence of Ubc4 protein, compared to the chromosomal extra copy. For both plasmids, Sup45 overproduction was confirmed by protein analysis (Figure 5C and 5D). Ability of the extra-copy of *SUP45* to ameliorate polyglutamine toxicity was abolished by a deletion of 19 C-terminal amino acids (Figure 5E), that impairs Sup45 function in translation and interaction with Sup35 [46], or by the missense mutation *sup45-103*, T62C (Figure 5F) that also impairs Sup45 function in translation termination [47]. Thus, Sup45 ability to ameliorate toxicity depends on the same sequence elements that control its function in translational machinery.

### Aggregation patterns of polyQ and release factors in the wild-type and *AQT* strains

**Figure 6:**
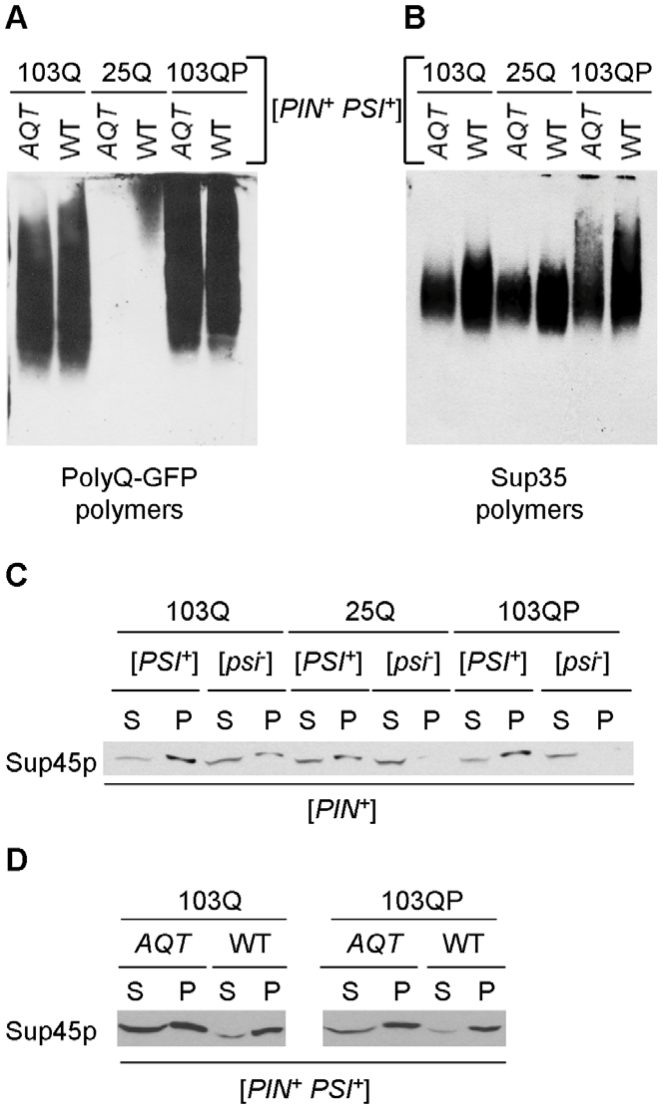
Effects of *AQT* on aggregation of release factors. A and B – Fractionation of the polyQ/QP-GFP (A) and Sup35 (B) polymers by sizes in the *ubc4*Δ [*PSI^+^*] strains either with (*AQT*) or without (WT) *AQT*. Polymers were separated by semi-denaturing agarose gel electrophoresis (SDD-AGE, see Text S1). Filter obtained from one and the same gel was reacted to either GFP (A) or Sup35C (B) antibodies. Polyglutamines alter distribution of Sup35 polymers, and this effect is counteracted by *AQT*. Experiment has been repeated with 3 independent cultures per each combination, each time with the same result. C – Expression of 103Q promotes aggregation of Sup45 protein (Sup45p) in the *ubc4*Δ [*PIN^+^ psi^−^*] strain, and expression of either 103Q or 103QP increases aggregate-associated fraction of Sup45 in the *ubc4*Δ [*PSI^+^*] strain, as detected by an increase in the pellet (P) versus supernatant (S) fraction in comparison to the respective strain expressing 25Q. Centrifugation was followed by Western blotting and reaction to the Sup45 antibody. D – Proportion of soluble (supernatant, S) versus aggregate-associated (pellet, P) Sup45 protein is significantly increased in *AQT ubc4*Δ [*PSI^+^*] strain, compared to the identical non-*AQT* (WT) strain, as determined by centrifugation analysis, followed by Western blotting and reaction to the Sup45 antibody.

As both polyglutamines and prion form of Sup35 form SDS-resistant polymers in the yeast cells, we have checked if patterns of their aggregation are influenced by the presence of an extra copy of *SUP45*. Both 103Q and 103QP proteins exhibit a broad range of distribution of the SDS-resistant polymers by size, as demonstrated by semi-denaturing agarose gel electrophoresis (SDD-AGE), with 103QP containing more protein in the higher molecular weight (MW) fraction (Figure 6A). This result confirms that the aggresome, formed by 103QP, contains insoluble protein aggregates, in contrast to the juxtanuclear quality control compartment (JUNQ) observed in the yeast cells with a defect of the ubiquitin-proteasome system [48]. Neither 103Q nor 103QP polymer distribution was significantly affected by *AQT* (Figure 6A). In the wild type [*PSI^+^*] cells containing non-expanded polyglutamines (25Q), Sup35 prion polymers were distributed within a relatively narrow range of sizes (Figure 6B). However, in the presence of either 103Q or 103QP, size range of the Sup35 polymers was increased and higher molecular weight (MW) polymers were accumulated, suggesting that some Sup35 could be associated with 103Q (or 103QP) polymers, therefore partly following their distribution. Notably, the Sup35 polymer size range became narrower in the presence of *AQT*, and the high MW fraction, which depends on 103Q/QP, disappeared (Figure 6B). This suggests that the extra dosage of Sup45 somewhat counteracts the increase in size of the Sup35 polymers and possibly, their interaction with polyglutamines.

We have also checked effects of polyglutamines and gene dosage on patterns of Sup45 aggregation. Sequestration of Sup45 by the Sup35 prion aggregates is known to contribute to cytotoxicity of overproduced Sup35 in [*PSI^+^*] strains [49]. We could not detect aggregate-associated Sup45 by SDD-AGE (data not shown), apparently because it is not converted into an amyloid form and is therefore released after SDS treatment. However, centrifugation analysis demonstrated that presence of either [*PSI^+^*] prion or 103Q protein resulted in the shift of a fraction of Sup45 protein to the pelletable (aggregate-associated) form, with both prion and 103Q together having an additive effect (Figure 6C). 103QP did not exhibit any observable effect on Sup45 aggregation in the [*psi^−^*] strain, however it further increased Sup45 aggregation in the presence of [*PSI^+^*]. Remarkably, proportion of the pelletable versus soluble Sup45 was decreased in the *AQT* (disomic) [*PSI^+^*] strain expressing 103Q or 103QP, compared to the identical strain not possessing disomy (Figure 6D). This showed that an increase in Sup45 levels counteracted its sequestration by aggregates.

Overall, our data indicate that both release factors, Sup35 and Sup45, are sequestered by polyglutamine aggregates in the [*PSI^+^*] cells, and that excess Sup45 not only restores supply of functional Sup45 but also changes the mode of Sup35 aggregation.

### Polyglutamines and translational readthrough

As our data point to sequestration of release factors as a mechanism of polyglutamine toxicity, we have checked if polyglutamines increase translational readthrough of stop codons. For this purpose, the chimeric constructs bearing a stop codon between the *PGK1* and *lacZ* ORFs have been employed. Surprisingly, no increase in translational readthrough (measured by β-galactosidase activity) has been detected in the presence of 103Q (Table S3). One possible explanation of these data is that damage to translational machinery, caused by the aggregation and sequestration of release factors in the presence of polyglutamines, is so severe that translation is arrested and not proceeding beyond the stop codon. Another (but not mutually exclusive) possibility is that cytotoxicity is related to non-translational functions of Sup35/45. Indeed, it has been reported that the immediate consequence of the severe shortage of a release factor in yeast is not translational defect per se, but cytoskeleton damage leading to cell death [50].

## Discussion

### Prion role in polyglutamine toxicity

Our data demonstrate that mechanism of polyglutamine toxicity depends on the prion composition of the cell. In fact, it appears that polyglutamine protein is not the toxicity agent itself, but rather amplifies the effects of the endogenous prion aggregates by sequestering them and making them more rigid. In case of Rnq1 prion, sequestration of Rnq1 by polyglutamines also leads to sequestration of the cytoskeletal proteins, interacting with Rnq1, and subsequent impairment of endocytosis [39], [40]. In this case, toxicity is relieved by re-localization of polyglutamines to an aggresome that removes polyQ (and possibly Rnq1) from the endocytic sites [42]. However, in case of Sup35 prion, relocalization is not sufficient for amelioration of toxicity. This could be due to the fact that Sup35 itself is an essential protein, and/or due to its normal distribution all over the cytoplasm, making it impossible to define specific toxicity sites. Additive action of Rnq1 and Sup35 prions on 103Q toxicity in the absence of the P-rich region also confirms that their cytotoxic effects are at least partly independent of each other.

Remarkably, our data show for the first time that the aggresome is not always cytoprotective. Moreover, it is possible that formation of the aggresome in the cell containing an essential protein in the form of self-perpetuating amyloid (prion) is itself cytotoxic due to sequestration of this essential protein. However, it remains uncertain whether toxicity is primarily driven by sequestration of Sup35 into an aggresome, or by its sequestration into the smaller polyglutamine aggregates remaining in the cytoplasm. While some Sup35 is definitely detected in the aggresome (Figure 1D), we don't know if the functional fraction of Sup35 is sequestered there. Moreover, while it is obvious that some fraction of Sup35 should retain function in the [*PSI^+^*] strain, as elimination of Sup35 is lethal [51], it remains unknown whether this functional component of Sup35 is represented by residual non-aggregated Sup35, smaller oligomers, or both. However, it is more likely that a fraction of oligomeric Sup35 remains functional, as amount of monomeric Sup35 retained by the strong [*PSI^+^*] strains, that is essentially at the limit of detection, seems too low for maintaining viability. Indeed, Sup35C regions are not included in the amyloid core and may stay enzymatically active, so that only size, composition and/or location of the aggregate would modulate its functionality in the cellular context. Changes in the distribution of Sup35 polymers by size in the presence of polyglutamines (Figure 6B), clearly show that certain alterations of Sup35 aggregation patterns, making them more similar to poly-Q aggregation patterns, coincide with toxicity.

### Modulation of polyglutamine toxicity by Sup45 dosage

Amelioration of [*PSI^+^*]-dependent cytotoxicity by extra-dosage of the Sup35 functional partner, Sup45, confirms that toxicity results from sequestration of release factor(s) by polyglutamine aggregates. Possibly Sup35, containing a QN-rich domain, is sequestered directly, while Sup45 is sequestered via its interaction with Sup35. Indeed, ability of polyglutamines to facilitate aggregation of endogenous QN-rich proteins even in a non-prion strain has been reported previously [43], [52] and confirmed by us (Figure 1E and Figure 6D), and it was shown that Sup35 prion aggregates produced at high levels cause toxicity via sequestering Sup45 [49]. In agreement with these data, *AQT* (i. e., extra copy of *SUP45*) ameliorates both polyglutamine toxicity (Figure 2) and toxicity of excess Sup35 (Figure 3C) in the [*PSI^+^*] cells. There is probably a competition for the Sup35/Sup45 complex between polyglutamine aggregates and functional sites (ribosome etc.) at which the Sup35/Sup45 complex is supposed to act. Therefore, an increased abundance of Sup45 not only increases a proportion of non-sequestered Sup45 but also partly counteracts sequestration of Sup35, that can be seen as a change in size distribution of the Sup35 polymers (Figure 6B). Hence, the antitoxic effect of excess Sup45.

### Role of *ubc4* deletion in *AQT* detection

The *AQT* derivatives were originally detected in the strain, lacking the major ubiquitin-conjugating enzyme Ubc4. One obvious reason for this is that amelioration of toxicity by *AQT* is more pronounced in the absence of Ubc4 (Figure 2F), making detection of the anti-toxic papillae easier. It is possible that Ubc4 promotes ubiquitination and subsequent degradation of a fraction of excess Sup45, that agrees with a more profound increase in Sup45 protein levels, which was detected in the *ubc4*Δ strain bearing an extra-copy of *SUP45*, in comparison to the isogenic *UBC4^+^* strain (Figure 4E). In addition, *ubc4*Δ may influence patterns of polyglutamine aggregation and/or ability of polyglutamines to sequester other proteins. Indeed, defects of the ubiquitin system are known to promote aggresome formation in mammalian cells [20], and *ubc4*Δ influences formation and aggregation of the [*PSI* ^+^] prion yeast, as well as levels of some Hsps and patterns of their interactions with prion aggregates [53].

Another, although not necessarily exclusive explanation of increased *AQT* appearance in *ubc4*Δ cells is that a lack of Ubc4 may affect chromosome segregation and/or recombination, therefore increasing the frequency of chromosome non-disjunction. Ubiquitination and ubiquitin-dependent protein degradation are involved in regulation of DNA repair and chromosome segregation [54], [55]. Ubc4 is implicated in ubiquitination of histones [56], and *ubc4*Δ is shown to affect proper segregation of some yeast plasmids [57]. Persisting variations of the chromosome II size in *AQT* strains (for example, see Figure S2B) and occasional appearance of weak additional bands on the CHEF gels of the *ubc4*Δ strains (data not shown) speak in favor of a detrimental effect of *ubc4*Δ on chromosome stability. It is possible that the presence of the foreign DNA (*KanMX* insertion) on chromosome II of the *ubc4*Δ strain aids in destabilization of this specific chromosome. Irrespective of the mechanism of the *ubc4*Δ effect, this deletion is required for neither maintenance of the chromosome II extra copy nor toxicity amelioration. The *UBC4^+^* strains with an extra copy of chromosome II were obtained by genetic cross and dissection and continued to maintain a disomy (data not shown), and amelioration of toxicity by excess Sup45 was still detected in the *UBC4^+^* strain, at a lower level in case of chromosome II extra copy (Figure 2F) and at more profound level for the plasmid-mediated excess Sup45 that is less sensitive to the presence of Ubc4 (Figure 5).

### Relevance of yeast data to human polyglutamine disorders

Involvement of translational machinery in HD has been suspected from some results in mammalian systems [58]. It remains unknown if polyglutamines can sequester the human homologs of Sup35 and Sup45 (respectively, eRF3 and eRF1), as mammalian ortholog of Sup35 does not have a QN-rich domain. However, our results could be relevant to mammalian systems in a more general way. About 40% of the variation in the age of HD onset, in cases where the polyglutamine repeat is of the same length, is due to DNA variation [59]. Our work provides a potential explanation for such a variation by demonstrating that changes in the abundance of the sequestered protein(s), occurring via alteration of either gene dosage or gene expression, can modulate polyglutamine toxicity. A non-DNA component of variations in polyglutamine toxicity can be explained by differences in the composition of other aggregated proteins (*e. g.* endogenous self-perpetuating aggregates or prions) present in the cell. Our results show that prion composition of the cell not only drives polyglutamine toxicity but also determines a pathway via which polyglutamines influence cell physiology, as proteins already associated with the other aggregates are more likely to be sequestered by polyglutamines. Mammalian cells contain a variety of proteins with the prion-like QN-rich domains, and machinery for propagation of the QN-rich protein aggregates exists in mammals [60]. Protein aggregation can also be induced by oxidative damage and other stresses. It was reported that artificially generated β-rich aggregates may sequester other proteins [61]. It is therefore entirely possible that organisms or tissues (or both) differ by the aggregate composition, and this in turn influences their susceptibility to polyglutamine disorders. Composition of endogenous aggregates may also modulate which proteins are sequestered by polyglutamines, as proteins associated with other aggregates interacting with polyglutamines are more likely to be sequestered, like Sup45 in the cells containing the Sup35 prion. This could explain why different groups are coming out with different conclusions in regard to both mechanisms of polyglutamine toxicity and contributions of different types of polyglutamine aggregates.

## Materials and Methods

### Yeast strains and growth conditions

The *Saccharomyces cerevisiae* strains, used in this study and listed in Table S4, are derivatives of GT81 series [62] of the prototype haploid genotype *ade1 his3 leu2 lys2 trp1 ura3*, with different mating types and various prion compositions. The individual gene deletions were made by using PCR-mediated transplacement with the cassette bearing either *Schizosaccharomyces pombe HIS5* gene, an ortholog of *S. cerevisiae HIS3* gene (thus designated in this paper as *HIS3*), or bacterial *kan^r^* gene, which causes resistance to G418 in yeast [63]. Spontaneous *AQT* mutants were initially obtained in the strain GT349 (*MAT* **a** *ubc4*Δ*::HIS3* [*PIN^+^ PSI^+^*]), as described in Results.

Standard yeast media, procedures (including transformation, phenotype scoring, velveteen replica plating, mating and sporulation), and growth conditions were used [64]. Yeast cultures were grown at 30°C except for the temperature-sensitivity assays (employing 39°C). Tetrad dissection was performed by using the MSM System 300 micromanipulator from Singer Instrument Co. Ltd. Analysis of yeast chromosomes by CHEF (Contour-clamped Homogeneous Electric Field) is described in Text S1.

Polyglutamine toxicity was detected as growth inhibition on the synthetic dropout medium with galactose instead of glucose where polyglutamine constructs were selectively maintained and induced. As most of our polyglutamine constructs were expressed from plasmids bearing the *URA3* marker, the plasmid-selective galactose medium (-Ura/Gal) was used, unless stated otherwise. Polyglutamine toxicity becomes more evident after longer incubation periods, as also confirmed by growth curves (see Figure 2C). Typically, velveteened plates were scanned following 5–10 days of incubation after a second passage on galactose medium, while spotted from solution (without dilutions) plates were scanned after 3–5 days, and serial decimal dilutions spotted onto galactose medium were scanned after 2–3 weeks.

### Plasmids

Major plasmids used in this study are described in Text S1. A list of plasmids is available in Table S5.

### Construction of chromosomal deletions

Strategy of making chromosomal deletions is described in Text S1.

### Fluorescence microscopy

Fluorescence microscopy was performed according to standard techniques, as described in Text S1.

### Protein analysis

Protein isolation and electrophoresis are described in Text S1. Semi-Denaturing Detergent-Agarose Gel Electrophoresis (SDD-AGE), used to fractionate the SDS-resistant protein polymers according to their sizes, was performed according to the standard protocol [65] with slight modifications. Proteins were diluted in 2% SDS, incubated for 5 min at room temperature before loading, run in the 1.5% agarose gel with 0.1% SDS in 1X TAE buffer containing 0.1% SDS, transferred to nitrocellulose membrane (Whatman) by capillary blotting, and reacted to appropriate antibody. Assay for β-galactosidase activity was performed according to the standard protocol [66], except that cell debris was removed by centrifugation to avoid light scattering before the OD reading at 420 nm was taken.

### Antibodies

Description of antibodies used in this study can be found in Text S1.

### DNA microarray analysis

Gene copy number was determined by hybridization to the complete DNA microarray of the *Saccharomyces cerevisiae* genome, as described previously [67]. Detailed information can be found in the Text S1.

## Supporting Information

Figure S1 *SUP45*-independent phenotypes associated with *AQT*. A – *ubc4*Δ causes complete inhibition of growth at 39°C. *AQT* partly compensates for this defect of growth. Cultures were incubated in the liquid YPD medium for 1 day and serial decimal dilutions were spotted onto a YPD plate. B – Deletion of *SUP45* does not affect compensation of temperature resistance by *AQT*. C - The invasive growth phenotype is eliminated by *AQT* in a *SUP45-*independent manner. Cells were patched on a YPD plate and grown for 2 days. The plate was scanned before and after gentle wash under running water for 3 min. Similar effect of *AQT* was observed in the *UBC4^+^* strain (not shown). D – *AQT* slightly increases growth of the [*PIN^+^ psi^−^*] *ubc4Δ* strain in –Ura/galactose+raffinose medium in the presence of 103Q, as seen after relatively short periods of incubation. It is not known if this effect is specific to 103Q or is a consequence of the general increase in robustness of the *AQT* strain in these conditions. Cultures were grown in liquid –Ura/glucose medium for 1 day, and washed 3 times prior to the induction of 103Q in –Ura/galactose+raffinose medium, starting with the inocula of the same concentration. Serial decimal dilution were spotted onto –Ura/glucose medium after 24 hrs of growth. E – Deletion of the extra copy of *SUP45* gene does not eliminate the *AQT* effect on growth in the presence of 103Q in the [*PIN^+^ psi^−^*] strain, confirming that the molecular basis of this phenotype is different from the antitoxicity detected in the [*PSI^+^*] background.
(TIF)

Figure S2 Additional evidence for the association of *AQT* with the extra-copy of chromosome II. A – Tetrad analysis of the diploid heterozygous by both *AQT* and *met3*Δ (a centromere-linked marker on chromosome X) demonstrates that *AQT* is centromere-linked, as seen from low proportion of tetratypes (T) in comparison to parental (PD) and non-parental (NPD) ditypes (P<0.001). *AQT* is scored by growth on –Ura/Gal medium in the presence of 103Q plasmid, and *met3*Δ is scored by lack of growth in the absence of methionine (-Met). Similar results (not shown) were obtained after sporulating and dissecting diploids generated by mating the *AQT* strain to the isogenic strains of the opposite mating type, containing disruptions of the centromere-linked genes *met28* (chromosome IX) or *met14* (chromosome XI). For an explanation of tetrad types, see ref. [64]. B – Chromosome fractionation by CHEF (left), followed by Southern blotting (right) demonstrates the presence of the extra copy of chromosome II in all *AQT* derivatives. Chromosome II bands are indicated by arrows on the CHEF gel, and visualized by hybridization to the labeled fragment of *SSA3* gene (located on chromosome II) on Southern blot. Per each independent *AQT* derivative (designated as *AQT #2*, *AQT #7* and *AQT #9*) and wild-type control, two isolates are tested. An extra-band chromosome II was also co-inherited with *AQT* in meiosis (not shown). Notably, electrophoretic mobilities of duplicated chromosomes varied among *AQT* derivatives, and in one *AQT* derivative (#2) the difference was detected between two isolates. Variations in electrophoretic mobilities of chromosome II copies were also detected after meiosis of the *AQT*-containing diploids (data not shown). As all isolates contain a duplication of the whole coding material of chromosome II (see Figure 4B), variations in electrophoretic mobility are apparently due to repetitive non-coding elements or may reflect exchanges of material between non-homologous chromosomes.
(PPT)

Table S1 Mendelian inheritance of *AQT*. Each *AQT* strain was mated to the isogenic wild type (WT) *ubc4*Δ strain of the opposite mating type.
(DOC)

Table S2 Recombination test for allelism of *AQT* derivatives. * In parentheses are numbers of tetrads showing the respective ratio. ** One exceptional tetrad with 3∶1 ratio was recovered.
(DOC)

Table S3 UGA readthrough in the absence and presence of 103Q. Cultures were grown in -Ura -Trp glucose medium to early stationary phase. Cells were washed 3 times before being transferred to -Ura-Trp/galactose+raffinose medium for 24-hr induction. Three independent cultures were tested. Differences are not statistically significant (*P_Ho_*>0.05).
(DOC)

Table S4 Yeast strains. * Chromosome II disomics.
(DOC)

Table S5 List of plasmids.
(DOC)

Text S1 Supporting Materials and Methods. Plasmid constructions; construction of chromosomal deletions; fluorescence microscopy; protein analysis; antibodies; electrophoretic separation of yeast chromosomes; DNA microarray analysis. References [30], [42], [46], [47], and [67]–[80] are quoted.
(DOC)
