# Supplementary figures and images for: Polyglutamine Toxicity Is Controlled by Prion Composition and Gene Dosage in Yeast
Source: PLoS Genet. 2012 Apr 19;8(4):e1002634. doi: 10.1371/journal.pgen.1002634 (PMC3334884; doi:10.1371/journal.pgen.1002634)

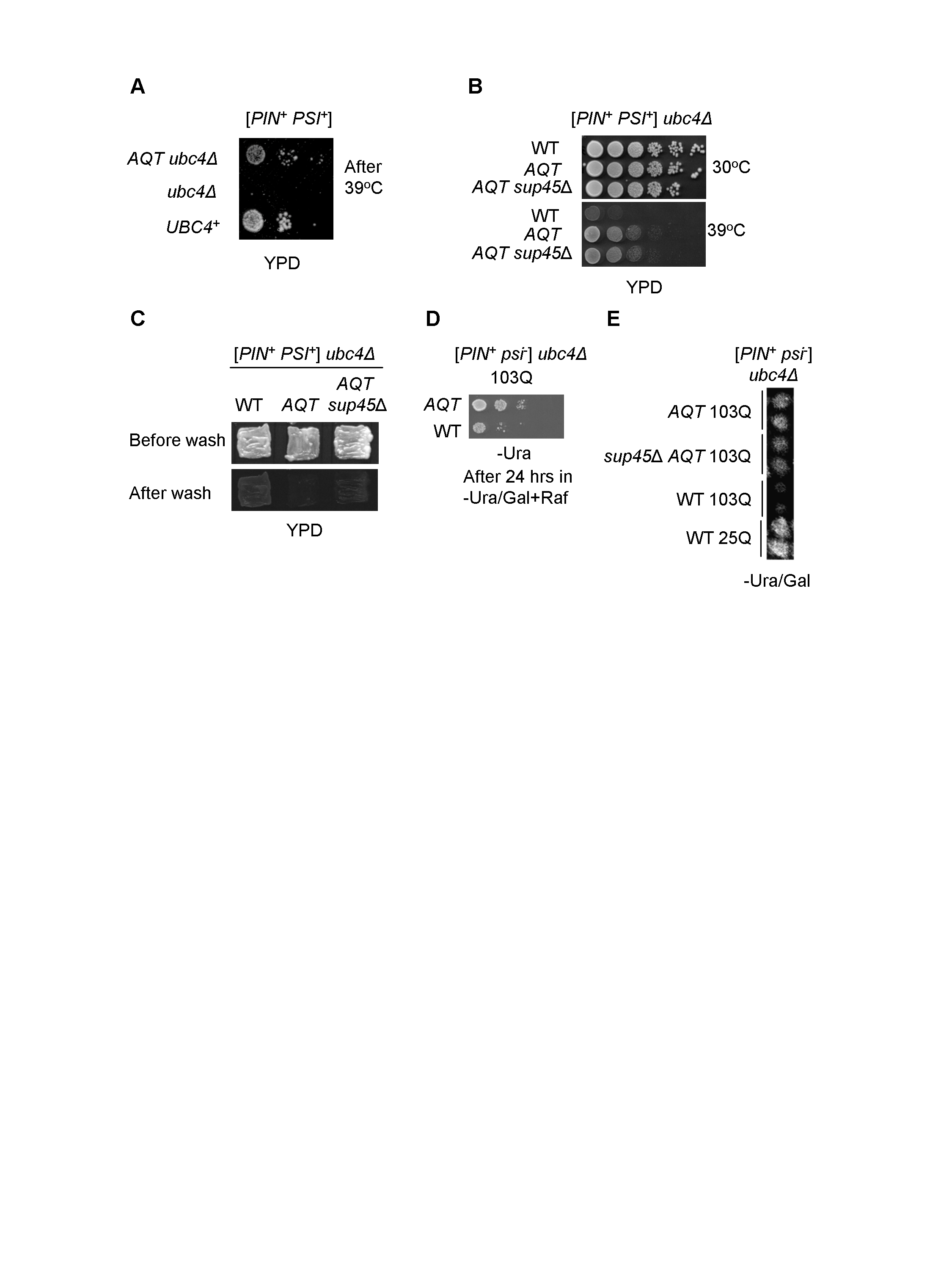

Supplement: Figure S1 — SUP45-independent phenotypes associated with AQT. A – ubc4Δ causes complete inhibition of growth at 39°C. AQT partly compensates for this defect of growth. Cultures were incubated in the liquid YPD medium for 1 day and serial decimal dilutions were spotted onto a YPD plate. B – Deletion of SUP45 does not affect compensation of temperature resistance by AQT. C - The invasive growth phenotype is eliminated by AQT in a SUP45-independent manner. Cells were patched on a YPD plate and grown for 2 days. The plate was scanned before and after gentle wash under running water for 3 min. Similar effect of AQT was observed in the UBC4+ strain (not shown). D – AQT slightly increases growth of the [PIN+ psi−] ubc4Δ strain in –Ura/galactose+raffinose medium in the presence of 103Q, as seen after relatively short periods of incubation. It is not known if this effect is specific to 103Q or is a consequence of the general increase in robustness of the AQT strain in these conditions. Cultures were grown in liquid –Ura/glucose medium for 1 day, and washed 3 times prior to the induction of 103Q in –Ura/galactose+raffinose medium, starting with the inocula of the same concentration. Serial decimal dilution were spotted onto –Ura/glucose medium after 24 hrs of growth. E – Deletion of the extra copy of SUP45 gene does not eliminate the AQT effect on growth in the presence of 103Q in the [PIN+ psi−] strain, confirming that the molecular basis of this phenotype is different from the antitoxicity detected in the [PSI+] background. (TIF) [file pgen.1002634.s001.tif]
