# Supplementary material for: Polyglutamine Toxicity Is Controlled by Prion Composition and Gene Dosage in Yeast
Source: PLoS Genet. 2012 Apr 19;8(4):e1002634. doi: 10.1371/journal.pgen.1002634 (PMC3334884; doi:10.1371/journal.pgen.1002634)
Supplement: Figure S2 — Additional evidence for the association of AQT with the extra-copy of chromosome II. A – Tetrad analysis of the diploid heterozygous by both AQT and met3Δ (a centromere-linked marker on chromosome X) demonstrates that AQT is centromere-linked, as seen from low proportion of tetratypes (T) in comparison to parental (PD) and non-parental (NPD) ditypes (P<0.001). AQT is scored by growth on –Ura/Gal medium in the presence of 103Q plasmid, and met3Δ is scored by lack of growth in the absence of methionine (-Met). Similar results (not shown) were obtained after sporulating and dissecting diploids generated by mating the AQT strain to the isogenic strains of the opposite mating type, containing disruptions of the centromere-linked genes met28 (chromosome IX) or met14 (chromosome XI). For an explanation of tetrad types, see ref. [64]. B – Chromosome fractionation by CHEF (left), followed by Southern blotting (right) demonstrates the presence of the extra copy of chromosome II in all AQT derivatives. Chromosome II bands are indicated by arrows on the CHEF gel, and visualized by hybridization to the labeled fragment of SSA3 gene (located on chromosome II) on Southern blot. Per each independent AQT derivative (designated as AQT #2, AQT #7 and AQT #9) and wild-type control, two isolates are tested. An extra-band chromosome II was also co-inherited with AQT in meiosis (not shown). Notably, electrophoretic mobilities of duplicated chromosomes varied among AQT derivatives, and in one AQT derivative (#2) the difference was detected between two isolates. Variations in electrophoretic mobilities of chromosome II copies were also detected after meiosis of the AQT-containing diploids (data not shown). As all isolates contain a duplication of the whole coding material of chromosome II (see Figure 4B), variations in electrophoretic mobility are apparently due to repetitive non-coding elements or may reflect exchanges of material between non-homologous chromosomes. (PPT) [file pgen.1002634.s002.ppt]

## Slide 1
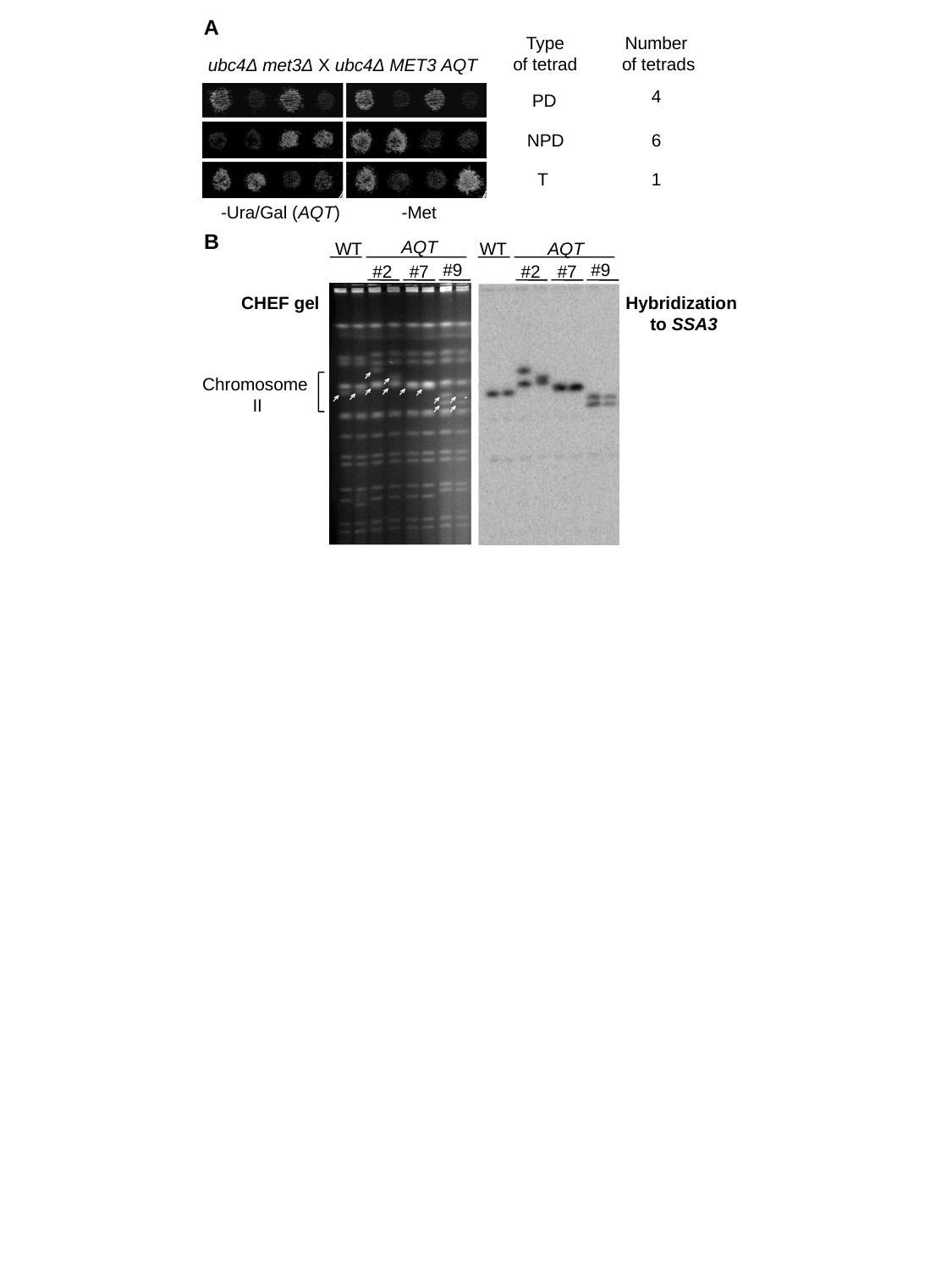

A
Type
of tetrad
Number
of tetrads
ubc4Δ met3Δ X ubc4Δ MET3 AQT
4
PD
NPD
6
T
1
-Ura/Gal (AQT)
-Met
B
AQT
WT
WT
AQT
#9
#9
#2
#7
#2
#7
CHEF gel
Hybridization
to SSA3
Chromosome
 II
