# Supplementary material for: Polyglutamine Toxicity Is Controlled by Prion Composition and Gene Dosage in Yeast
Source: PLoS Genet. 2012 Apr 19;8(4):e1002634. doi: 10.1371/journal.pgen.1002634 (PMC3334884; doi:10.1371/journal.pgen.1002634)
Supplement: Table S1 — Mendelian inheritance of AQT. Each AQT strain was mated to the isogenic wild type (WT) ubc4Δ strain of the opposite mating type. (DOC) [file pgen.1002634.s003.doc]

**Table S1. Mendelian inheritance of *AQT***

| *AQT* isolate | Numbers of tetrads with *AQT*:WT ratios | | | | | Total number of tetrads |
| --- | --- | --- | --- | --- | --- | --- |
|  | 4:0 | 3:1 | 2:2 | 1:3 | 0:4 |  |
| *AQT2* | 0 | 0 | 5 | 0 | 2 | 7 |
| *AQT7* | 0 | 0 | 10 | 0 | 3 | 13 |
| *AQT9* | 0 | 0 | 8 | 0 | 0 | 8 |
