# Supplementary material for: Polyglutamine Toxicity Is Controlled by Prion Composition and Gene Dosage in Yeast
Source: PLoS Genet. 2012 Apr 19;8(4):e1002634. doi: 10.1371/journal.pgen.1002634 (PMC3334884; doi:10.1371/journal.pgen.1002634)
Supplement: Table S2 — Recombination test for allelism of AQT derivatives. * In parentheses are numbers of tetrads showing the respective ratio. ** One exceptional tetrad with 3∶1 ratio was recovered. (DOC) [file pgen.1002634.s004.doc]

**Table S2. Recombination test for allelism of *AQT*** derivatives

| Crosses  (all are [*PIN^+^ PSI^+^*] *ubc4*Δ) | *AQT*: WT ratios^*^ | Total number of tetrads analyzed |
| --- | --- | --- |
| *AQT2* X *AQT7* | 4:0 (6)^**^ | 7 |
| *AQT2* X *AQT9* | 4:0 (8) | 8 |
| *AQT7* X *AQT9* | 4:0 (9) | 9 |
