# Supplementary material for: Polyglutamine Toxicity Is Controlled by Prion Composition and Gene Dosage in Yeast
Source: PLoS Genet. 2012 Apr 19;8(4):e1002634. doi: 10.1371/journal.pgen.1002634 (PMC3334884; doi:10.1371/journal.pgen.1002634)
Supplement: Table S3 — UGA readthrough in the absence and presence of 103Q. Cultures were grown in -Ura -Trp glucose medium to early stationary phase. Cells were washed 3 times before being transferred to -Ura-Trp/galactose+raffinose medium for 24-hr induction. Three independent cultures were tested. Differences are not statistically significant (PHo>0.05). (DOC) [file pgen.1002634.s005.doc]

**Table S3. UGA readthrough in the absence and presence of 103Q**

| Poly-Q | Readthrough, % |
| --- | --- |
| 25Q | 0.43 ± 0.07 |
| 103Q | 0.32 ± 0.15 |
