# Supplementary material for: Polyglutamine Toxicity Is Controlled by Prion Composition and Gene Dosage in Yeast
Source: PLoS Genet. 2012 Apr 19;8(4):e1002634. doi: 10.1371/journal.pgen.1002634 (PMC3334884; doi:10.1371/journal.pgen.1002634)
Supplement: Table S4 — Yeast strains. * Chromosome II disomics. (DOC) [file pgen.1002634.s006.doc]

**Table S4. Yeast strains**

| Strain | Genotype | Reference |
| --- | --- | --- |
| GT81-1C | *MAT***a** *ade1 his3 leu2 lys2 trp1 ura3* [*PIN^+^ PSI^+^*] | [62] |
| GT159 | *MAT***a** *ade1 his3 leu2 lys2 trp1 ura3* [*PIN^+^ psi^-^*] | [35] |
| GT409 | *MAT***a** *ade1 his3 leu2 lys2 trp1 ura3* [*pin^-^ psi^-^*] | [53] |
| GT563 | *MAT***a** *ade1 his3 leu2 lys2 trp1 ura3 rnq1*Δ*::HIS3*  [*pin^-^ PSI^+^*] | This study |
| GT81-1D | *MAT***α** *ade1 his3 leu2 lys2 trp1 ura3* [*PIN^+^ PSI^+^*] | [62] |
| GT349 | *MAT***a** *ade1 his3 leu2 lys2 trp1 ura3 ubc4*Δ*::HIS3*  [*PIN^+^ PSI^+^*] | [53] |
| GT386 | *MAT***a** *ade1 his3 leu2 lys2 trp1 ura3 ubc4*Δ*::HIS3*  [*PIN^+^ psi^-^*] | [53] |
| GT574* | *MAT***a** *ade1 his3 leu2 lys2 trp1 ura3 ubc4*Δ*::HIS3/ubc4*Δ*::HIS3* [*PIN^+^ PSI^+^*] | This study |
| GT532-9B* | *MAT***α** *ade1 his3 leu2 lys2 trp1 ura3 ubc4*Δ*::HIS3/ubc4*Δ*::HIS3* [*PIN^+^ PSI^+^*] | This study |
| GT1203 | *MAT***a** *ade1 his3 leu2 lys2 met3*Δ*::KanMX trp1 ura3 ubc4*Δ*::HIS3* [*PIN^+^ PSI^+^*] | This study |
| GT1219 | *MAT***a** *ade1 his3 leu2 lys2 trp1 ura3 ubc4*Δ*::KanMX*  [*PIN^+^ PSI^+^*] | This study |
| GT1555* | *MAT***a** *ade1 his3 leu2 lys2 trp1 ura3*  *SUP45/sup45*Δ*::LEU2 ubc4*Δ*::HIS3/ubc4*Δ*::HIS3*  [*PIN^+^ PSI^+^*] | This study |
| GT1665-3A* | *MAT***a** *ade1 ubc4*Δ*::HIS3/UBC4 his3 leu2 lys2 trp1 ura3* [*PIN^+^ PSI^+^*] | This study |
