# Supplementary material for: Polyglutamine Toxicity Is Controlled by Prion Composition and Gene Dosage in Yeast
Source: PLoS Genet. 2012 Apr 19;8(4):e1002634. doi: 10.1371/journal.pgen.1002634 (PMC3334884; doi:10.1371/journal.pgen.1002634)
Supplement: Table S5 — List of plasmids. (DOC) [file pgen.1002634.s007.doc]

**Table S5. List of plasmids**

| Protein | Plasmid | Marker/Type | Promoter | Reference |
| --- | --- | --- | --- | --- |
| Sup35C | pRS315-SUP35del3ATG | *LEU2/CEN* | *SUP35* | This study |
| LacZ | pUKC815 | *URA3/*2μ | *PGK* | [72] |
| LacZ w/UGA | pUKC819 | *URA3/*2μ | *PGK* | [72] |
| Htt-25Q-GFP | pYES-Q25trp | *TRP1/*2μ | *GAL1* | [42] |
| Htt-103Q-GFP | pYES-Q103trp | *TRP1/*2μ | *GAL1* | [42] |
| Htt-25Q-GFP | pYES2-25Q | *URA3/*2μ | *GAL1* | [30] |
| Htt-25QP-GFP | pYES2-25QP | *URA3/*2μ | *GAL1* | [42] |
| Htt-103Q-GFP | pYES2-103Q | *URA3/*2μ | *GAL1* | [30] |
| Htt-103QP | pYES2-103QP | *URA3/*2μ | *GAL1* | [42] |
| Ubc4 | pTRP-UBC4 | *TRP1/*2μ | *GAL1* | [72] |
| Sup35NM- DsRed | pCUP-Sup35NM-DsRed | *LEU2/CEN* | *CUP* | This study |
| Sup45ΔC5 | pRS315- Sup45ΔC5 | *LEU2/CEN* | *SUP45* | This study |
| Sup45 | pRS315-GAL- SUP45cDNA | *LEU2/CEN* | *GAL1* | This study |
| Sup45 | pRS315-SUP45 | *LEU2/CEN* | *SUP45* | [68] |
| Sup45-103 | pRS315-SUP45-103 | *LEU2/CEN* | *SUP45* | [47] |
| Sup45 | pRS316-SUP45 | *URA3/CEN* | *SUP45* | [68] |
| Sup45ΔC19 | pDB843-SUP45-ΔC19 | *LEU2/CEN* | *SUP45* | [46] |
