# Supplementary material for: Polyglutamine Toxicity Is Controlled by Prion Composition and Gene Dosage in Yeast
Source: PLoS Genet. 2012 Apr 19;8(4):e1002634. doi: 10.1371/journal.pgen.1002634 (PMC3334884; doi:10.1371/journal.pgen.1002634)
Supplement: Text S1 — Supporting Materials and Methods. Plasmid constructions; construction of chromosomal deletions; fluorescence microscopy; protein analysis; antibodies; electrophoretic separation of yeast chromosomes; DNA microarray analysis. References [30], [42], [46], [47], and [67]–[80] are quoted. (DOC) [file pgen.1002634.s008.doc]

**Polyglutamine toxicity is controlled by prion composition and gene dosage in yeast**

He Gong, Nina V. Romanova, Kim D. Allen, Pavithra Chandramowlishwaran, Kavita Gokhale, Gary P. Newnam, Piotr Mieczkowski, Michael Y. Sherman and Yury O. Chernoff

**Supporting Materials and Methods**

**Plasmid constructions.** Plasmids used in this study are listed in Supporting Table S5. Poly-Q/QP producing constructs were described previously . Centromeric plasmids bearing wild type or mutant (*sup45-103*) alleles of *SUP45* gene under its endogenous promoter were a gift of G. Zhouravleva (St. Petersburg State University, Russia). Plasmid CEN-SUP45ΔC19, bearing the deletion of 19 C-terminal codons in *SUP45*, that impairs protein function and its interaction with Sup35 , was a gift of D. Bedwell (University of Alabama at Birmingham). The galactose-inducible *SUP45* plasmid, containing the *SUP45* cDNA under control of the *P_GAL1_* promoter, was previously isolated in the Chernoff laboratory in a library screen aimed at amelioration of excess Sup35 toxicity. The CEN-SUP35C plasmid was constructed by inserting the *Pst*I-*Xba*I fragment, bearing the *SUP35C* region under the endogenous *SUP35* promoter, from plasmid pEMBL-yex-SUP35 del3ATG , into pRS315. The plasmid pCUP-Sup35NM-DsRed was constructed by D. Kiktev in the Chernoff laboratory via inserting the *Eco*RI-*Sac*II fragment, that contains the copper-inducible promoter (*P_CUP1_*) and NM domains of Sup35 from plasmid pmCUPNMsGFP , into the vector pRS315 cut at the corresponding sites, and fusing it in frame to the coding region of DsRed2 from pDsRed2-N1 (Clontech). The plasmid overexpressing *UBC4* was constructed by inserting the *UBC4* ORF into the position between the *GAL1* promoter and *CYC1* terminator in the plasmid pTRP . Plasmids carrying the promoter and N-terminal domains of the yeast *PGK* gene, fused to the bacterial β-galactosidase (*lacZ*) gene either in frame or via a UGA stop codon , were a gift of M. Tuite (University of Kent).

**Construction of chromosomal deletions.** Chromosomal deletions in the extra copy of chromosome II were made in two consecutive steps via PCR-mediated genome alteration. The first step involved the insertion of a cassette containing the *URA3* gene of *Kluyveromyces lactis* and *E. coli hph* (hygromycin B phosphotransferase) gene into the targeted regions as counterselectable markers ; the second step employed the replacement of targeted regions with the *LEU2* gene amplified from vector pRS315 . Resulting Ura^-^ Leu^+^ hygromycin sensitive transformants were checked by PCR to confirm that they contained the deletions.

**Fluorescence microscopy**. GFP and/or DsRed visualization in live cells was typically performed after 6 hours of induction in liquid media containing 2% galactose and 2% raffinose, and/or 200 μM CuSO_4_, depending on the promoter(s) used. Fluorescence was detected under a BX41 microscope (Olympus) with the endow GFP bandpass emission (green) or tetramethyl rhodamine isocyanate (rhodamine/Dil; red) filters.

**Protein analysis**. For protein isolation, cells were destroyed by vortexing with glass beads in the lysis buffer (25 mM Tris-HCl, pH 7.5; 0.1 M NaCl; 10 mM EDTA; 4 mM PMSF; 200 μg/ml Cycloheximide; 2 mM Benzamidine; 20 μg/ml Leupeptin; 4 μg/ml Pepstatin A; 1 mM NEM; Roche complete protein inhibitor cocktail of suggested dose), and cleared of cell debris at 735 g. Protein concentrations were measured by Bradford reagent (BioRad) and, if necessary, verified by Coomassie staining of the SDS-PAGE gels. Differential centrifugation experiments employed 8,000 g for 10 min at 4°C. Pellets were resuspended in the volume of lysis buffer that was equivalent to the volume of supernatant. Protein samples were always boiled prior to loading onto SDS-PAGE. After electrophoresis, proteins were transferred onto Hybond ECL nitrocellulose membrane (GE Healthcare) and reacted to appropriate antibodies. Reaction was detected by using the chemiluminescent detection reagents as described in the GE Healthcare protocols. Densitometry was performed to determine relative protein levels using the VisionWorks LS program from UVP (UVP, LLC, Upland, CA) as described previously .

**Antibodies**. Rabbit polyclonal antibodies to Sup35C and Sup45 were a gift of D. Bedwell (University of Alabama at Birmingham). Rabbit anti-GFP antibody was purchased from Sigma-Aldrich. Antibody to Ade2 protein was described earlier . Secondary anti-rabbit HPR antibody, used in immunoblotting, was purchased from Sigma-Aldrich.

**Electrophoretic separation of yeast chromosomes.** Yeast chromosomes were separated by using Bio-Rad CHEF (Contour-clamped Homogeneous Electric Field) Mapper XA following the protocol described previously . Chromosome II was identified by Southern blotting, followed by hybridization to the P^32^ labeled *SSA3* probe.

**DNA microarray analysis.** In this study, we used custom made microarrays , which consisted of PCR products amplified from S288C genomic DNA printed on polylysine coated glass slides. This version of the microarrays contains about 13000 different PCR probes. These probes offer nearly complete coverage of the *S. cerevisiae* genome . The specific coordinates of probes with links to their annotation in the *S. cerevisiae* genome can be found at the site (http://www.ncbi.nlm.nih.gov/projects/geo/query/acc.cgi?acc=GPL4414). The procedures used to prepare, label and hybridize genomic DNA for Comparative Genomic Hybridization (CGH) arrays were described previously .

The hybridization signal was collected with a GenePix 4000B scanner and GenePix Pro 6.0 software (Molecular Devices). The resulting data files were deposited in the University of North Carolina Microarray Database (http://genome.unc.edu), and tools available at that site were used to transform the raw data (normalized Log2 Red/Green ratios according to the median). The transformed data from experimental arrays and from four control hybridizations (Cy3-reference strain DNA x Cy5-reference strain DNA) were analyzed with CGH-Miner software. This software is using the Clusters Along Chromosomes method to identify DNA copy number alterations in CGH-arrays .
